# Supplementary material for: Patient’s experience with the Arabin cervical pessary during pregnancy: A questionnaire survey
Source: PLoS One. 2022 Jan 12;17(1):e0261830. doi: 10.1371/journal.pone.0261830 (PMC8754293; doi:10.1371/journal.pone.0261830)
Supplement: S1 File — (DOCX) [file pone.0261830.s001.docx]

| 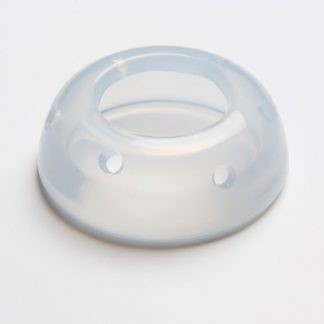 | *Dear Ms.*  *Sorry to bother you, but we are evaluating our previous patients who had a cervical pessary to prevent preterm birth, and we would like to ask you 10 questions. To answer them, you might need no more than 5 minutes. We would be grateful to receive your answers within the next 2 weeks. You will find enclosed an envelope that you can use if you send it back by post mail.*  *You may also scan it and send it back by email to the following mail address: …*  *Thank you for your cooperation!* |
| --- | --- |

1) Did you have all information and advice that you needed before insertion of the pessary?

- Yes
- No

2) An increase in vaginal discharge is the most common side effect of pessary treatment: did the physician tell it to you before cervical insertion?

- Yes
- No

3) Did you experience an increase in vaginal discharge?

- Yes
- No

*If yes, please explain on a scale of 1-10 whether this bothered you, if necessary please explain:_________________________________________________________________________*

4) Did you have any other side effects during the pessary treatment ?

- Yes
- No

5)Did the pessary treatment change your daily life?

- Yes, positively
- Yes, negatively
- No

6)Did you have any discomfort during the treatment with a pessary?

- Yes
- No

7)Did you think that the follow-up that you received was adequate ?

- Yes
- No

8) How did the treatment meet your expectations?

- [Better than I expected](https://context.reverso.net/traduzione/inglese-italiano/better+than+I+expected)
- [Worse than I expected](https://context.reverso.net/traduzione/inglese-italiano/better+than+I+expected)
- [As I expected](https://context.reverso.net/traduzione/inglese-italiano/better+than+I+expected)

9)Was the removal of the pessary painful?

- Yes
- No

*If yes, please explain on a scale from 1-10 the degree of pain_______________*

10)In a similar situation would you chose the pessary treatment again or recommend it to a good friend?

- Yes
- No

Thank you for your help
